# 

**Published:** 1890-12

**Authors:** 


### BOOKS AND PAMPHLETS RECEIVED.

Jahresbericht der Gesellschaft fur Natur und Heildkunde in Dresden, 1890.

Verhandlungen der Naturhistorischen Vereines, Bonn. 1889–1890.

Bulletin Mensuel Societé Linneenne du Nord de la France. Ameins.

Bulletin of the American Museum of Natural History. Vol. III, No. I. New York, 1890.

Annalen des K. K. Naturhistorischen Hofmuseums. Redigirt von Dr. Franz Ritter von Hauer. Bd. V, No. 3, Wein, 1890.

Bollettino dei Musei di Zoologia ed Anatomia comparata della R. Universita di Forino, Vol. V, Nos. 74 to 86.

Anales de la Sociedad Ciéntifica Argentina. Tome XXX, Ent. 1 and 2. Buenos Ayres, 1890.

Sitzungsberichte der Naturforscher-Gesellschaft bei der Universitat. Dorpat, 1890.

Sitzungsberichte der Physikalischemedicinischen Gesellschaft zu Wurzburg, Nos. 6 and 7. 1890.

Bulletin de la Société Zoologique de France. Paris, 1890.

Proceedings of the Linnean Society of New South Wales. Vol. IV, pl. 4; Vol. V, Pt. 1. Sidney, 1890.

Ornis Jahr. VI. Heft. 2 and 3. Wein, 1890.

Il Naturalista Sicileano. Palermo.

Mittheilungen des Ornithologischen Vereines in Wein. Vol. XIV, No. 16 and 17.

Thiermedizinische Vortrage. Herausgegeben von Dr. George Schneideniuhl. Bd. II, Heft. 2. Leipzig. Arthur Felix, 1890.

The Blood and Blood Vessels in Health and Disease, by Wesley Mills, M. A., M. D. Rep. from N. Y. Med. Jour., Sept. 13th, 1890.

The Treatment of the Morphine Disease, by J. B. Mattison, M. D. Rep. from The Therapeutic Gazette, 1890.

The Relation of Bacteria to Practical Surgery, by John B. Roberts, A. M., M. D.

Bulletin of Illinois State Laboratory of Natural History. Vol. III, Art. 5–10. Champaign.

Journal and Proceedings of the Royal Society of New South Wales. Vol. XXIII, Pt. 2. Sydney.

Proceedings Colorado Scientific Society. Vol. III, Pt. 2. Denver.

Journal Gincinnati Society of Natural History. Vol. XIII, No. 3.

Bollettino Scientifico. Pavia.

Jahres-Bericht der Schlesischen Gesellschaft fur Naturlandische Cultur. Breslan, 1890.

Sitzungsbericht der Mathematisch-Physikalischen Classe der K. B. Akademie der Wissenschaften zu Muchen, 1890, Heft. 1–3.

Natuurkundig Tydschrift voor Nederlandsch-Indie, utgegeven door de Koninklijke Natuurkundige Vereeniging. Deel XLIX. Batavia, 1890.

Bulletin de la Société Vondoise des Sciences Naturelles. Lausanne, 1890.

Societé Linneenne der Nord de la France. Nos. 219–20. Amiens.

Anales de la Sociedad Cientifica Argentina. Tomo. XXX, Ent. 3–4. Buenos Ayres.

Der Zoologische Garten. Frankfort.

Il Naturalista Siciliano. Palermo.

Johns Hopkins University Circulars. Baltimore.

